# Impact of oxytosis on the cross‐talk of mTORC with mitochondrial proteins in drug‐resistant cancer stem cells

**DOI:** 10.1002/jcp.31421

**Published:** 2024-08-26

**Authors:** Santhi L. Pandrangi, Prasanthi Chittineedi, Ram K. Manthari, Balaji Suhruth

**Affiliations:** ^1^ Department of Life Sciences, School of Science GITAM (Deemed to be) University Visakhapatnam India

**Keywords:** calcium, mitochondria, mTOR, oxytosis

## Abstract

By delivering the environmental inputs to transport nutrients and growth factors, Mechanistic Target of Rapamycin (mTOR) plays a significant role in the growth and metabolism of eukaryotic cells through the regulation of numerous elementary cellular processes such as autophagy, protein synthesis, via translation of mitochondrial protein transcription factor A mitochondrial, mitochondrial ribosomal proteins, and mitochondrial respiratory complexes I &V that are encoded in the nucleus with the help of translation initiation factor 4E–BP. These mitochondrial proteins are involved in cell signaling to regulate proper cell growth, proliferation, and death which are essential for tumor growth and proliferation. This suggests that tumor cells are dependent on mTORC1 for various metabolic pathways. However, this crucial regulator is activated and regulated by calcium homeostasis. Mounting evidence suggests the role of calcium ions in regulating mitochondrial enzymes and proteins. Hence, disrupting calcium homeostasis leads to calcium‐dependent cell death called “Oxytosis” through hampering the expression of various mitochondrial proteins. “Oxytosis” is a novel non‐apoptotic cell death characterized by glutamate cytotoxicity and ferritin degradation. The present review focuses on the crosstalk between mTORC1 and mitochondrial proteins in the cancer pathophysiology and the impact of calcium ions on disrupting mTORC1 leading to the induction of “Oxytosis.”

## 1 INTRODUCTION

Globally, cancer is the prime cause of death. According to a cancer survey, there are about 2.25 million active cancer cases in India as of 2021, and around 1.2 million cases increase annually. Cancer is a cellular impairment disorder characterized by a loss of regulation to control a cell's growth, proliferation, and death (Aldinucci et al., 2020; Ozaki and Nakagawara, 2011). It should be noted that cell death is as important as cell growth and development as it regulates the generation of new cells. The phenomena where cells lose their ability to die associated with a gain in ability to proliferate is the hallmark of cancer, leading to cell accumulation (Lakhanpal et al., 2016).

Traditional treatment strategies, such as chemotherapy and radiotherapy, coupled with surgical removal, clear bulk tumor cells leaving behind cancer stem cells (CSCs). CSCs can be defined as a small subpopulation of cells present in the quiescent stage of cell cycle with the characteristic normal stem cell‐like properties (NSCs), capable of re‐initiating tumors and are believed to be the major cause of tumor relapse/recurrence (Pandrangi et al., 2014a). by Bonnet and Dick while working on acute myeloid leukemia patients, observed a few cells exhibiting self‐renewal ability, differentiation potential, and enhanced proliferation and named them as CSCs (Kuşoğlu and Biray Avcı, 2019; Pandrangi et al., 2014b). Although conventional therapies target tumor cell death through induction of apoptosis/DNA damage, none of them are potent in targeting CSC death. This is because these cells are in the quiescent phase and escape from all the therapies (Kruyt and Schuringa, 2010). The presence of CSC makes cancer patients more vulnerable to death, leading to a higher mortality rate and worse survival outcomes (Gulati et al., 2021; Kumar et al., 2013). Hence, new approaches that specifically target bulk cancer cells and CSCs are to be discovered to enhance the survival outcome and free from tumor relapse/recurrence. CSCs can be usually identified by the presence of certain surface markers such as CD44, CD24, CD133, ALDH, etc. since, CSCs are present in the quiescent state, these cells are not exposed to the drugs or radiation making these cells resistant. Additionally, these cells will be expressing the DNA repair proteins which help them to repair the DNA damaged as a result of exposure to drugs or radiation. Hence, CSCs are also characterized with therapy resistance.

**Figure 1:**
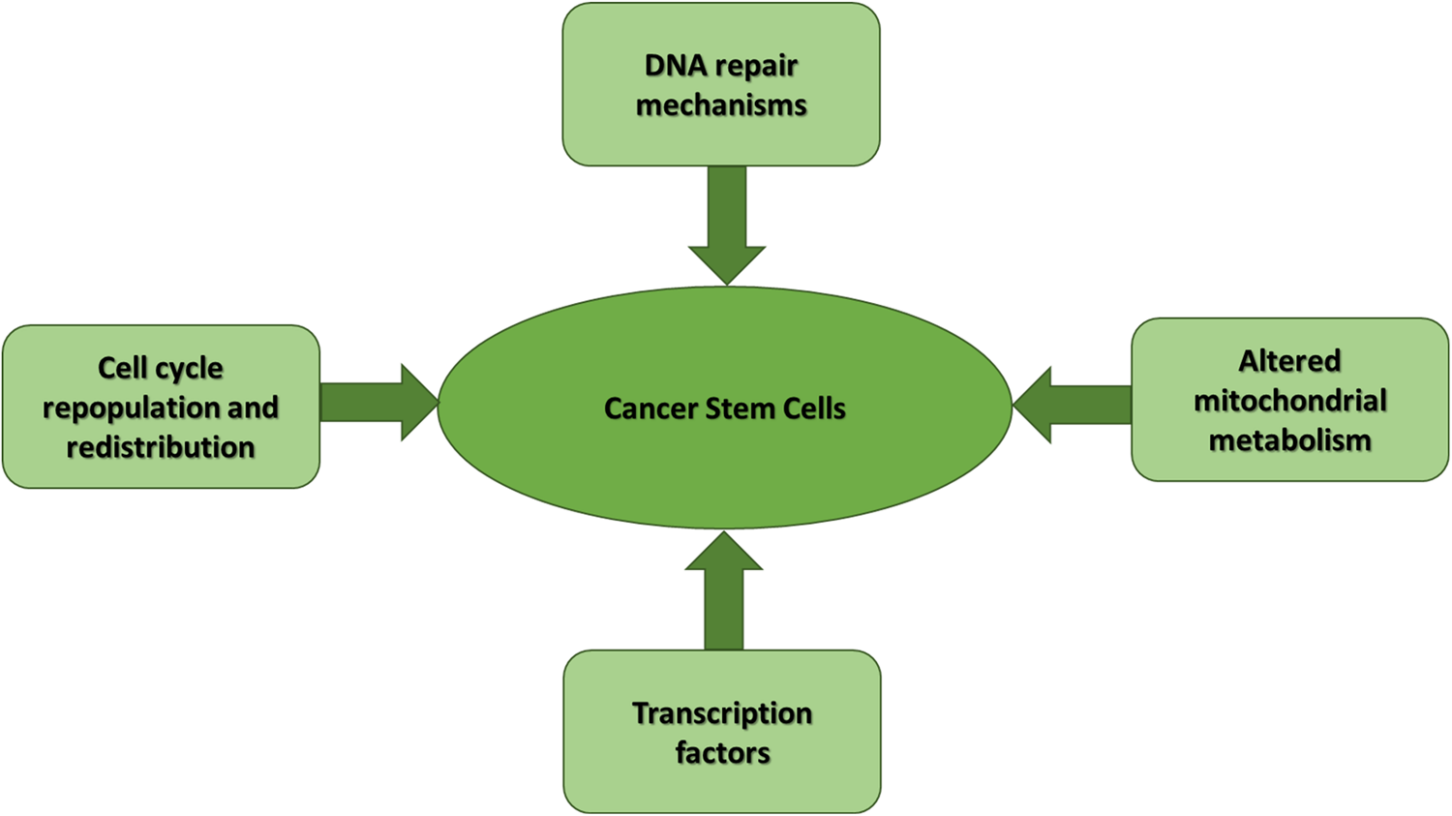
Various factors contributing tumor stemness.

The growth, proliferation, and death processes are regulated by numerous proteins, which, when mutated, transform normal cells into malignant tumor cells (Chittineedi et al., 2022a; Mahboobnia et al., 2021). Mitochondria, regarded as a powerhouse of cells, harbors various proteins involved in cell cycle regulation. Apart from that, it is well known that tumor cells rely upon a bulk ATP source, which is obtained from mitochondria (Morita et al., 2013). This suggests that mitochondria and their associated proteins have a vital role in the transformation of pre‐cancerous lesions to cancer and subsequent metastasis. Transcription factor A mitochondrial (TFAM), mitochondrial ribosomal proteins, and mitochondrial respiratory complexes I and V are a few mitochondrial proteins that are the critical drivers of tumor cell progression due to their potent role in the cell cycle. Figure 1 depicts the role of TFAM and mitochondrial protein in regulating the cell cycle. However, these proteins are dependent on Mechanistic Target of Rapamycin (mTOR). mTOR a PI3K‐related kinase family provides various nutrients and growth factors that are essential for eukaryotic cellular growth and metabolism. It is one of the critical regulators of numerous essential cellular processes like protein synthesis and autophagy (Saxton and Sabatini, 2017). Disrupted mTOR signaling could be considered an early sign of tumor progression, the aging process, or diabetes (Rambatla et al., 2021). mTOR contains f two unique protein complexes in their catalytic subunit termed as mTORC1 and mTORC2. mTORC1 in turn has 3 principal components: (i) mTOR (ii) Raptor‐regulatory protein. Raptor binds to the TOR signaling motif thereby facilitating the recruitment of its substrate to mTORC1. (iii) mammalian lethal with Section 13 protein 8 (MLST8) – It is also known as GβL. mLST8 gets associated with the mTORC1, and it stabilizes the kinase activation loop. In addition to these, it also encompasses 2 inhibitory subunits DEP domain containing mTOR interacting protein (DEPTOR) and proline‐rich Akt substrate of 40 kDa (PRAS40) (Ramanathan and Schreiber 2009). Activation of mTORC results in overexpression of tumor hypoxia‐inducing factors resulting in the proliferation of cells to the G1 phase of the cell cycle, leading to enhanced angiogenesis and cell division, respectively (Morita et al., 2015). The present review focuses on the cross‐talk between the mToRC1 signaling pathway and the role of various mitochondrial proteins that mTORC regulates in the pathophysiology of various malignancies.

## 2 MITOCHONDRIA AND MTORC CROSS TALK

It is well evident that mTOR is associated with various elements in mitochondria, particularly with the mitochondrial outer membrane. In addition, it has been shown that in the presence of mitochondrial inhibitors, the activity of mTORC1 has been drastically reduced (Carter and Hood, 2012). Supporting this, a study claimed that the presence of mTOR‐complex characterizes Jurkat T cells, the mitochondrial fraction; this complex is strongly associated with mitochondrial activity, especially with elevated mitochondrial membrane potential, consumption of O_2_, as well as with increased ATP production capacity (de la Cruz López et al., 2019). Voltage‐dependent anion channels (VDACs), on the other hand, are the pore‐forming proteins localized on the outer mitochondrial membrane, which serve as the binding sites for various cytoplasmic enzymes, including glycerol kinase and hexokinase isoforms, allowing preferential access to mitochondrial ATP (Varughese et al., 2021). This suggests that mTOR regulates the synthesis of mitochondrial ATP via VDACs and rapamycin a potent mTOR inhibitor treatment results in co‐precipitation of mTOR with VDAC1 and BCL‐xl a key mediator that regulates mitochondrial function and cellular apoptosis (Baines et al., 2007). The list of various mTOR inhibitors for cancer therapy are listed in Table 1.

**Table 1:** List of mTOR inhibitors under clinical trials.

Inhibitor name	Generation	Clinical trials conducted on
RAD001	First	Advanced RCC
Rapamycin	First	Acute renal allograft
CCI‐779	First	Advanced RCC
INK128	Second	Advanced solid tumors
OSI027	Second	Lymphoma
AZD8055	Second	Glioma/HCC
Rapalinks	Third	No clinical trials

Abbreviation: HCC, hepatocellular carcinoma cells; mTOR, Mechanistic Target of Rapamycin.

Also, numerous studies have demonstrated that mTORC1 facilitates the translation of mitochondrial proteins involved in cell signaling that are encoded in the nucleus with the help of translation initiation factor 4E–BP. The major mitochondrial proteins regulated by mTOR are TFAM, Mitochondrial ribosomal proteins, and mitochondrial respiratory complexes (Sepulveda‐Villegas et al., 2020). These proteins play an important role in the regulation of the cell cycle. For instance, TFAM plays a prominent role in the cell cycle in the G1/S phase by activating transcription at two major sites LSP and HSP1. Meanwhile, mitochondrial ribosomal proteins are associated with proteins like cyclin B1/Cdk1 during the cell cycle (Pérez‐Plasencia et al., 2019). They function as riboproteins, which are essential for the translation of mitochondrial mRNAs. They play a prominent role during the transition of a cell from the G2 to the M phase. On the other hand, mitochondrial respiratory complex I (ubiquinone oxidoreductase) is involved in the electron transport chain and is responsible for the oxidation of NADH, while mitochondrial respiratory complex V (ATP Synthase) is involved in oxidative phosphorylation, which catalyzes the synthesis of ATP in bulk.

Since mitochondria are considered to be the “Powerhouse” of the cell, bulk tumor cells and CSCs require mitochondria abundantly, hence, any process that mediates mitochondrial generation serves as a critical regulator for tumor initiation, progression, as well as invasion. Mitochondrial fusion/fission and mitochondrial biogenesis are two among them. Mitochondrial fusion/fission represents two opposite functions; the former one forms network, and the latter one forms fragments. Optic atrophy 1 (OPA1), dynamin‐related GTPases, and mitofusin (MFN) are a few genes that confer mitochondrial fusion and fission (Al Ojaimi et al., 2022). Recent findings demonstrate that drug‐resistant cancer cells or CSCs elevate OPA1 and/or MFN1/2 to enhance the expression of cytochrome C. Similarly, when gallbladder cells were exposed to gemcitabine, these cells induced mitochondrial fusion by the activation of MCL1 protein as a survival mechanism (Wang et al., 2021).

Mitochondrial biogenesis is also one of the crucial events that is linked with drug resistance in CSCs. This process is involved in regulating mitochondrial mass, quality, and its function, clearing up unfunctional mitochondria, and synthesizing new mitochondria. This complex process is regulated by PGC‐1α which is found to be associated with tumor drug resistance. Under environmental stress, PGC‐1α induces mitochondrial biogenesis as well as oxidative phosphorylation leading to tumor survival and metastasis. Previous findings from Liu et al. (2020) and Suomalainen and Nunnari (2024) demonstrated that inhibiting mTORC or targeting mitochondrial biogenesis sensitizes melanoma cells to MAPK inhibitors. These authors demonstrated that melanoma cells with either N‐Ras or B‐Raf mutations confer resistance to MEK inhibitors (Liu et al., 2020; Suomalainen & Nunnari, 2024).

Mitochondrial biogenesis and mitophagy, a programmed eliminating process of excess mitochondria through its degradation, are also involved in maintaining the mitochondrial quality. PTEN‐induced putative kinase 1 (PINK1)‐Parkin pathway is considered one of mitophagy's classical/specific receptors. Other receptors of mitophagy include FUN14 domain‐containing protein 1 (FUNDC1), BNIP3, BCL‐2‐like protein 13(BCL2L13). Interestingly, these receptors are found to enhance tumor proliferation as well as confer resistance to conventional drugs such as doxorubicin, cisplatin, paclitaxel, and 5‐fluoro‐uracil through mitophagy (Popov, 2020). Hong Wu et al. (2020) observed that under hypoxia‐induced sorafenib resistance in hepatocellular carcinoma cells (HCC), PINK‐1‐driven mitophagy was hyperactivated suggesting that suppressing mitophagy might sensitize HCC (Wu et al., 2020). Similar results conferring drug resistance through PINK‐1‐mediated mitophagy were observed in esophageal, breast, and lung carcinoma.

## 3 SIGNIFICANCE OF MITOCHONDRIAL PROTEINS IN CELL SIGNALING

Mitochondria regulate their own signaling in two ways: they serve as corporeal platforms on which protein‐protein‐signal communications take place, and they regulate the level of signaling molecules such as intracellular reactive oxygen species (ROS) and Ca^2+^ (Pandrangi et al., 2022a; Tait & Green, 2012; Terry et al., 2002). Mitochondria are therefore involved in governing of various cellular processes, including growth factor signaling, proliferation, and differentiation (Finkel et al., 2015). The regulated cellular death process is essential for the maintenance of proper homeostasis and tissue development of all organisms, and its dysregulation could contribute to numerous, including neurodegeneration and cancer (Elmore, 2007). The elementary form of regulated cellular death is apoptosis, a mechanism that is mediated by caspase protease (CP) activation. Upon caspase activation, they degrade various proteins which ultimately leads to rapid apoptotic cell death coupled with morphological changes such as plasma membrane blebbing (late‐stage apoptosis) (Enari et al., 1998; Pandrangi et al., 2022a). Mitochondria are involved in various mechanisms that regulate caspase activity thereby promoting apoptosis in multicellular organisms (Tait and Green, 2012). TFAM is one of the mitochondrial proteins that plays a prominent role in the stimulation of mitochondrial DNA replication and transcription leading to mitochondrial biogenesis (Hsieh et al., 2021). TFAM plays an important role in cell cycle regulation. It critically regulates the transition of a cell from the G1 phase of the cell cycle to the S phase (Roy et al., 2022). Since tumor cells tend to proliferate abruptly it is evident that TFAM is regarded as an oncogene and is highly expressed in malignant cells.

## 4 ROLE OF MITOCHONDRIAL PROTEINS IN CANCER

(Elmore, 2007; Enari et al., 1998; Finkel et al., 2015; Pandrangi et al., 2022a; Tait and Green, 2012; Terry et al., 2002; Hsieh et al., 2021; Roy et al., 2022) Nuclear‐encoded mitochondrial proteins such as Tfb2, TFAM, and mitochondrial RNA polymerase (Polrmt) are decoded into the cytosol and encrypt a mitochondrial target sequence, permitting their import into the mitochondria, where their function is regulated (Chittineedi et al., 2023; Kang et al., 2018). Key electron transport chain (ETC) proteins (e.g., cytochrome b subunits and ND) are translated in mitochondrial DNA (mtDNA), and because these proteins are further translated into the matrix of mitochondria, coordinated induction of mitochondrially encoded ribosomal RNA (rRNA) and transfer RNA (tRNAs) expression is also required (Skibinski et al., 2017). The whole mechanism is extremely sensitive to nutrient availability, redox stress, and mitochondrial function. It is to be noted that defects in mitochondrial biogenesis lead to embryonic mortality and disease (Boland et al., 2013; Handy & Loscalzo, 2012; Pandrangi et al., 2022b). Mitochondrial mass elevates with respect to the cellular size ratio, although it varies depending on mitochondrial biogenesis and is tightly regulated (Tait and Green, 2012). Table 2 summarizes the localization of various mitochondrial proteins.

**Table 2:** Represents the localization of various mitochondrial proteins regulated by mTOR.

Mitochondrial proteins regulated by mTOR	Localization
TFAM	Cytosol
TfB2	Cytosol
Polmrt	Cytosol
Sub units of Cytochrome‐C	Mitochondrial genome

Abbreviations: mTOR, Mechanistic Target of Rapamycin; TFAM, Transcription factor A mitochondrial.

Various oncogenes and tumor suppressors regulate mitochondrial biogenesis. Interestingly, mitochondrial biogenesis is stimulated by the c‐Myc oncogene via inducing PGC‐1β expression, resulting in enhanced expression of important mitochondrial proteins, including TFAM, Polγ, and NRF (Morrish & Hockenbery, 2014). Many cancers have somatic mtDNA mutations and precise immune response to mutated mitochondrial proteins has been observed. Interestingly, one could target mitochondria in cancer cells to elicit an adaptive immune response against mutated mitochondrial proteins.

Mitochondrial dysfunction is characterized by respiratory defects, membrane potential loss, Fe‐S cluster synthesis defects, mitochondrial degraded protein response (UPRmt), and altered genome expression assisted by numerous mechanisms. For instance, defective Fe‐S complex synthesis might lead to genome instability. It is evident that the release of cytochrome c governs mitochondrial dysfunction and leads to tumor‐suppressor‐mediated cellular death, scientifically termed apoptosis (Malla et al., 2018). Most mitochondrial proteins, including proteins necessary for mtDNA expression, and a few components of ETC complexes are transcribed in the genomic DNA and further translated into ribosomes localized in the cytosol and transmitted by mitochondria through peptides that serve as appropriate signals. These mitochondrial proteins are more governed by mTORC1. Because mTORC1 regulates cell proliferation, most energy might be consumed during mTORC1 regulation, suggesting that mTORC1 responds to bioenergetic variations, a process regulated by mitochondria. In addition, mTORC1 has been shown to regulate ATP synthesis via mitochondria and tumor cell progression. Differences in the mtDNA sequence can lead to changes in mitochondrial proteins, mostly ETC components, affecting their activity, ETC efficiency, and ROS production (Zhou et al., 2020).

Circumvention of cellular death is one of the major pathological events that provide biological benefits and is associated with tumor cell survival (Latha Pandrangi et al., 2022); in this scenario, however, the mitochondrial proteins that contribute to tumor markers perform various biological activities during homeostasis. For instance, under normal homeostasis, members of the BCL2 family, such as BAX, BCL2, and DIABLO, facilitate protein transportation. However, dysregulation of their expression levels results in tumor resistance in these cells (Pandrangi et al., 2022c). Only a subset of mitochondrial proteins is actively involved in various malignancies or with elevated levels of mitochondrial proteins whose role has not been fully investigated.

## 5 ROLE OF MITOCHONDRIAL PROTEINS IN DRUG RESISTANCE, METASTASIS, AND STEMNESS

Multiple signaling pathways such as the AKT pathway, mTOR pathway, or PIK3 pathway could be capable of modifying/reprogramming the mitochondrial function, leading to enhanced tumorigenesis. For instance, the turnover of follicle adhesion, which is a key component of cell mobility, is dependent on mitochondrial potential to generate ATP. This suggests that the tumor cells, which require bulk ATP to proliferate in the hostile tumor microenvironment, modulate the mitochondrial functioning to enhance tumor cell motility, leading to metastasis. As we know, metastasis is the process of spreading the tumor from one region to another through the lymphatic system. Dario Altieri et al. observed that numerous gene products such as an anterograde motor (KIF5B), an adaptor protein (TRAK), a static anchor (SNPH), and atypical mitochondrial GTPases (RHOT1), which are linked to the trafficking of the cytoskeletal system of neuronal cells, are also associated with tumor cell invasion via localizing and aggregating mitochondria to the periphery of the invading tumor cell. This group identified alternatively spliced SNPH isoforms in metastatic tumor cells and elucidated the role of SNPH isoforms in regulating mitochondrial trafficking. The key findings from their study are that mitochondrial SNPH regulates bioenergetics and oxidative stress, leading to controlled mitochondrial trafficking and tumor cell mobility.

Investigations into the protein folding of organelles revealed that mitochondrial integrity is one of the key factors of tumor metastasis. Interestingly, the protein folding mechanism in mitochondria relies upon two branches; one is the heat shock protein‐90 (Hsp‐90) and TNFR‐associated protein‐1 (TRAP‐1) homology of Hsp‐90 refolding followed by AAA+ mediated proteolysis such as ClpP of misfolded and aggregated proteins. Both these branches function complementarily and are overexpressed in various cancers. Seo JH et al. (2016) demonstrated that ClpP gene silencing halted tumor cell motility and suppressed metastasis in mice. This is due to low energy bioavailability in tumor cells as a result of mitochondrial misfolding.

CSCs are the subpopulation of tumor cells that are capable of initiating tumors and are genotypically like NSCs with the potential to self‐renew, differentiate, etc. Each CSC might exhibit a different metabolic profile from other CSCs and is associated with therapy resistance due to quiescence. It is evident that CSCs express very little reactive nitrogen and oxygen species (RNOS) levels inside their cells to maintain self‐renewal potential and sustain the hostile tumor microenvironment, which could be possible due to mitochondrial reprogramming. Due to elevated RNOS, CSCs exhibited high resistance to DNA damage even after combined chemo/radiotherapy sessions, suggesting that maintenance of RNOS is also a key feature for regulating therapy resistance mediated by the generation of oxidative stress. Dysregulation of ATP‐Binding Cassette (ABC) transporter members is also associated with drug resistance. ABCB6, ABCB7, ABCB8, and ABCB10, among all ABC transporters, are synthesized in mitochondria, suggesting the role of mitochondrial proteins in elevating therapy resistance in tumors leading to the development of therapy resistance in CSCs (Batlle & Clevers, 2017) (Figure 1).

## 6 ROLE OF CALCIUM IONS IN CELLULAR GROWTH, PROLIFERATION, AND DEATH

Intracellular calcium ions ([Ca^+2^]_i_) serve as a major regulator that regulates normal cell survival, growth, differentiation, metabolism, proliferation, and so on. It serves as a secondary messenger for several enzymes involved in the functioning of nerve cells, immune cells, gene activation, cell proliferation, apoptosis induction, cell cycle progression, etc. Although the cytoplasmic calcium ions [Ca^+2^]_C_ are very low, stored calcium levels in the Endoplasmic reticulum (ER) is 10,000 folds higher and is stored in the form of calsequestrin.

Not only as a secondary messenger, Ca^+2^ is also involved in cell proliferation stimuli at its early stage via activation of growth factors such as tyrosine‐kinase‐associated receptors, G protein‐linked receptors, etc. It is known that cellular proliferation is directly linked with cell cycle progression, suggesting the importance of Ca^+2^ in the cell cycle. In human T lymphocytes, Ca^+2^, along with calmodulin (CaM) regulates the expression of various cyclin‐dependent kinases (CDKs) such as CDK1, CDK2, as well as cyclin B.

To maintain cellular homeostasis, mitochondria form dynamic connections with neighboring intracellular organelles via calcium signaling. MAM, the mitochondrial‐associated ER membrane plays a prominent role in Ca^+2^ uptake and is shown to correlate with drug resistance (Chittineedi et al., 2022b; Huang et al., 2023). Mitochondrial Ca^+2^ uniporter complex (MCUC) comprising various subunits maintain mitochondrial Ca^+2^ homeostasis. Suppression of these subunits eventually restricted Ca^+2^ transport leading to the acquisition of drug‐resistance in cervical cancer cell lines HeLa. Hence, a detailed understanding of the importance of calcium homeostasis in mitochondria is essential in discovering novel therapeutic targets that can induce cell death in both bulk cancer cells and CSCs.

## 7 ROLE OF CALCIUM SIGNALING IN REGULATING MITOCHONDRIAL PROTEINS AND ITS IMPACT ON TUMOR PROGRESSION

It is evident that the mitochondria harbor bulk calcium ions which serve as the second messenger to regulate various metabolisms. Numerous studies suggest mitochondrial calcium accumulation results in ATP augmentation via calcium‐sensitive mitochondrial enzyme activity alteration. This suggests that since tumor cells have high demand for ATP supply, they tend to upregulate genes that are responsible for calcium uptake into mitochondria. Not only for mitochondrial regulation, calcium is also required to regulate mTORC1 (Romero‐Garcia et al., 2011). Studies by Ruo‐Jing Li et al. in 2016 demonstrated that calcium and calmodulin are essential metabolites that mTORC1 needs for its activation (Li et al., 2016). This suggests that calcium levels play a crucial role in regulating mitochondrial functioning, thereby enhancing cell proliferation directly through mitochondrial interaction as well as indirectly through mTORC activation.

**Figure 2:**
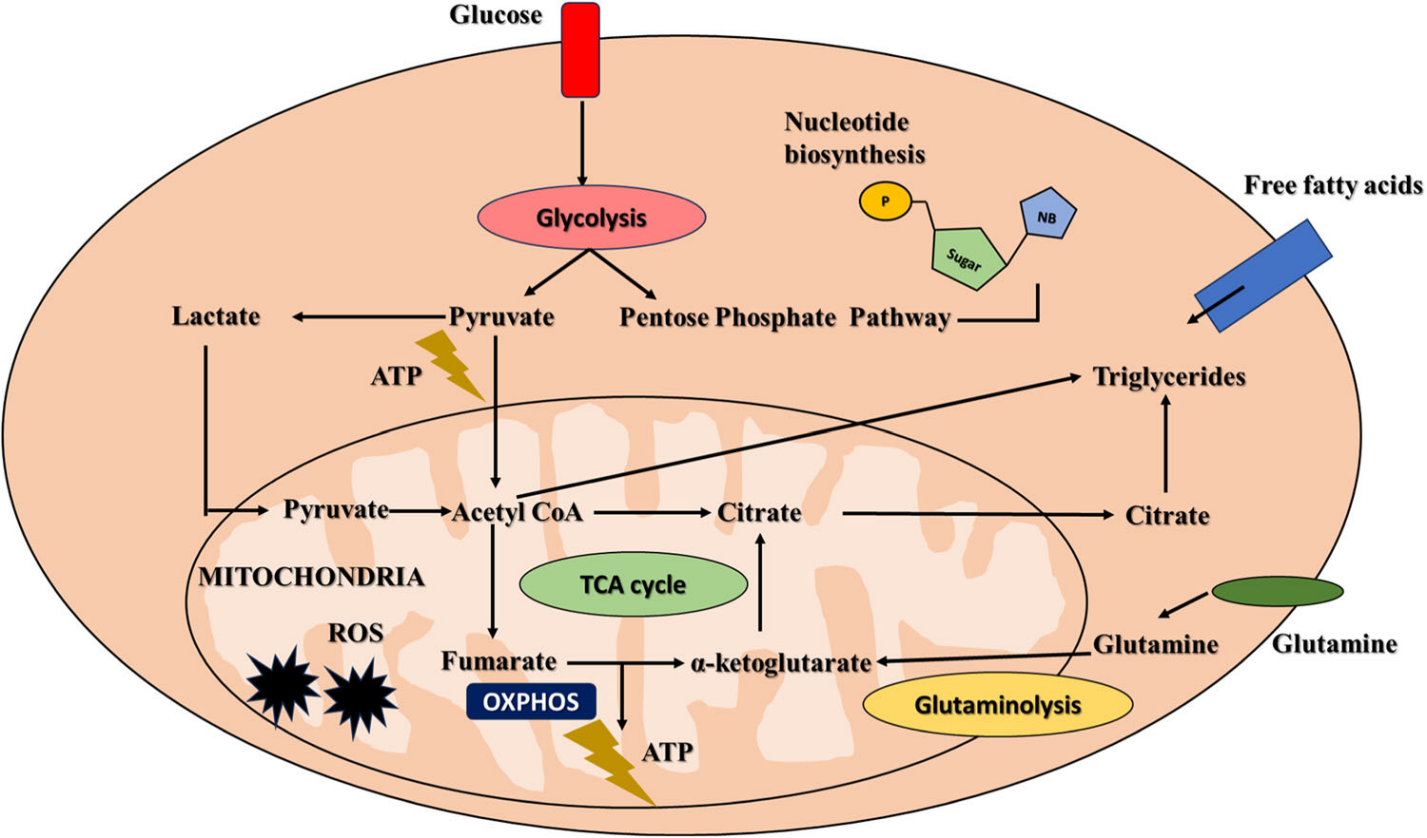
Metabolic functions of mitochondria. Mitochondria which is considered the powerhouse of the cells, is the master regulator for energy generation via glycolysis and the TCA cycle, as well as regulates glutaminolysis through fatty acid metabolism.

Calcium uptake into mitochondria targets enzymes of the TCA cycle. Although tumor cells exhibit Warburg's effect by bypassing the TCA cycle, it plays a prominent role in metabolizing a few important molecules, such as glutamine, that are essential for cell metabolism (Satheesh & Büsselberg, 2015). Glutamine is regarded as one of the key metabolites to protect tumor cells from cell death pathways and glutaminolysis, the process that generates glutamate via glutamate lysis. Glutamate is one of the crucial metabolites harbored by malignant cells to escape from cell death due to ROS production. In malignant cells, glutamate couples with cystine and glycine to form glutathione, which is regarded as a lipid peroxide scavenger molecule that catalyzes the conversion of lipid peroxides to lipid hydroxides (Pandrangi et al., 2022a). Interestingly, several oncogenes, such as c‐Myc, HIF, p53, and RAS, regulate tumor cell proliferation by utilizing the byproducts of the TCA cycle. For instance, as discussed earlier, glutaminolysis is a critical step to abscond cell death for tumor cells. C‐Myc, a proto‐oncogene that is upregulated in various malignancies, transcriptionally activates numerous genes and enzymes that regulate glutaminolysis through the upregulation of glutamate transporters, which is accompanied by enhanced glutamine import into the cell (Figure 2) (Miller et al., 2012). Hence, targeting calcium metabolism provides new insights into targeting tumor cell death.

## 8 VARIOUS MECHANISMS INVOLVED IN CALCIUM ABSORPTION AND SUBSEQUENT METABOLISM IN NORMAL CELLS

Serum Ca^+2^ levels are maintained by the collective homeostasis between the parathyroid glands, intestine, kidney, and bone. To maintain good bone health Ca^+2^ homeostasis is crucial. This is regulated by paracellular and transcellular pathways. Claudin 2, 12, and 15 which are regarded as tight junction (TJs) proteins are involved in the paracellular pathway. While in the transcellular pathway, Ca_v_1.3 and 4.1 R are the chief proteins involved. The paracellular pathway is regarded as a non‐saturable route, that maintains Ca^+2^ concentration in the intestine. The transcellular pathway on the other hand is a metabolically driven transport pathway, is dependent on concentration gradient and majorly involves mucosal‐serosal Ca^+2^ flux.

## 9 THE PARACELLULAR PATHWAY

The specialized membrane domains referred as tight junctions (TJs) primarily located in the apical portion of enterocytes, control the passage of small molecules and ions across this pathway. TJ are intercellular structures where the plasma membranes of neighboring cells are close to one another. Transmembrane proteins, cytoskeleton components, and cytoplasmic plaques make up these junctions. TJ structures' transmembrane proteins, which include occludin (Ocln) and claudins (Cldns), are generated in neighboring cells. These proteins bind to intercellular connections, preventing materials from freely moving through the paracellular space. Cldn 2, 12, and 15 oversee Ca^+2^ transport in the gut. Cldn 1 and Cldn 5 have diverse fastening activities that may influence Ca^+2^ transport through influencing general paracellular permeability. The role of Ocln in intestine Ca^+2^ absorption is still unknown. Ocln is a tetraspanin transmembrane protein, however, its precise function is unknown. Ca^+2^ transport across the TJ is a passive process that is influenced by the concentration and voltage gradient across the epithelium. When optimum or high Ca^+2^ intake prevails, it is predominantly transported into the jejunum and ileum. When Ca^+2^ intake is large, this route becomes essential because the sojourn time in the colon is brief and proteins implicated in the transcellular pathway are downregulated.

## 10 THE TRANSCELLULAR PATHWAY

The three steps that make up the transcellular pathway of duodenal Ca^+2^ absorption is the entry of Ca^+2^ across the enterocyte's brush border membranes (BBM) through Ca^+2^ channels of the epithelial cells; the movement of Ca^+2^ to the basolateral membranes (BLM) via BBM through high Ca2+ affinity (calbindins (CB)); and the extrusion of Ca^+2^ into the circulation via Ca^+2^‐ATPase localized to the plasma membrane. The epithelial Ca^+2^ channel transient receptor potential vanilloid 6 (TRPV6) and TRPV5 are the two most common epithelial channels involved in Ca^+2^ uptake by the enterocytes. The expression of these proteins is ubiquitously seen in the kidney and intestine. However, TRPV5 is seen in the intestine while TRPV6 is the isoform expressed in the kidney and is also detected in the colon as well as duodenum of humans, mice, and rats. TRPV6 is one of the most important participants in human intestinal Ca^+2^ absorption, however, its specific involvement remains unknown. TRPV6^‐/‐^ animals nonetheless transport a large quantity of Ca^+2^, indicating that additional channels or molecules contribute considerably to intestinal Ca^+2^ absorption. TRPV6 and TRPV5 are also found in the pancreas, prostate, mammary glands, sweat glands, and salivary glands. Both channels can be joined to create heterotetrameric channel complexes with unique characteristics and their expression is regulated by dietary calcium, calcitriol, and estrogen. Calbindins (CB) on the other hand, were traditionally recognized to carry Ca^+2^ to the basolateral membranes (BLM) of the cell from the apical side of an enterocyte. Apart from transporting Ca^+2^ CBs also serve to maintain intracellular Ca^+2^ concentrations, thereby protecting the cells from premature cell death mediated by apoptosis.

## 11 1,25 (OH)_2_D_3_‐MEDIATED INTESTINAL CALCIUM ABSORPTION

1,25 (OH)_2_D_3_ plays a crucial role in modifying enterocyte structure and function that leads to better intestinal Ca^+2^ absorption. 1,25 (OH)_2_D_3_ is known an active substituent of vitamin D. It is well known that vitamin D is obtained through sunlight, which should be further metabolized for its active functioning. Once the vitamin D is absorbed, it would be bound to the vitamin D response elements (VDE) which is mediated by the vitamin D receptor (VDR) located in the target genes via the cellular effects. The life of a third week VDR KO mice provides strong evidence about the role of VDR and its corresponding ligand in the intestinal calcium absorption. Although there were no distinguishing features between normal mice and VDR KO mice during birth, variations in growth patterns and mineral uptake homeostasis were observed in later stages of the life cycle. 10‐week‐old VDR KO mice showed drastic decrease in duodenal Ca^+2^ absorption accompanied with impaired TRPV6, TRPV5, and CB_9k_ expression suggesting that intestinal Ca^+2^ and vitamin D absorption are crucial were developmental process in rodents. Apart from regulating calcium homeostasis VDR is also critical regulator that is essential for cell proliferation, migration, as well as stress response mainly in the small intestine.

## 12 ROLE OF CALCIUM‐MEDIATED CELL DEATH

Calcium is required by both cancer cells and CSCs to regulate various metabolisms and to sustain in the hostile tumor microenvironment. It is evident that mitochondria are the harbor for calcium ions. Apart from the evidence of calcium levels in mitochondria, a few studies also demonstrated low levels of intracellular calcium ions (Park et al., 2009). Hence, calcium ion influx results in a mitochondrial impairment, which is also coupled with glutamate oxidation. Oxidation of glutamate makes it unavailable to synthesize glutathione, which ultimately leads to the accumulation of lipid ROS (Pandrangi et al., 2022d).

**Figure 3:**
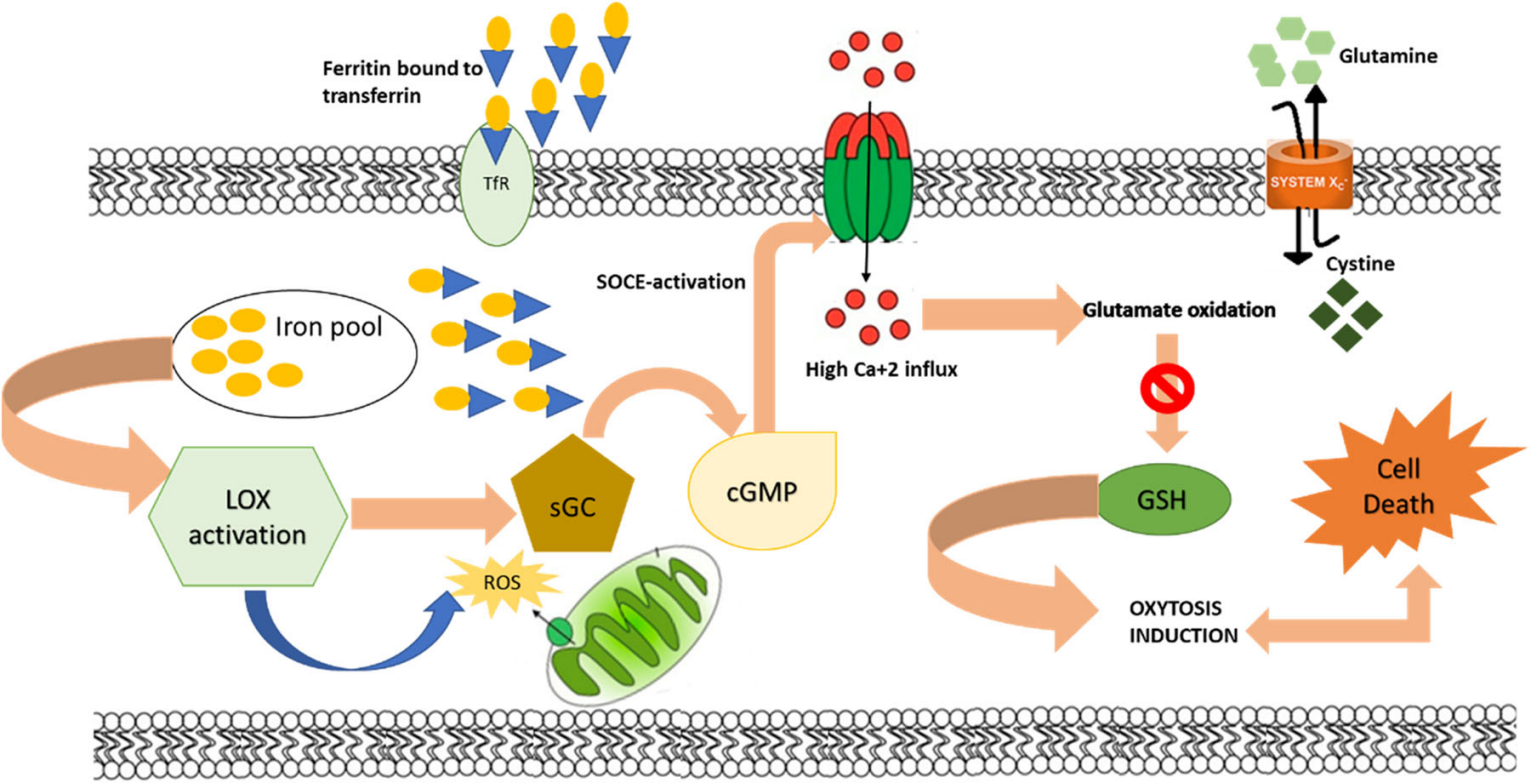
Mechanism of oxytosis. Oxytosis is a calcium regulated oxidative glutamate cytotoxicity resulted due to calcium accumulation into the cells. Under glutathione deprivation the cell enhances the expression of LOX which activates Store‐operated calcium entry channels (SOCE). SOCE further activates certain downstream calcium pumps which triggers calcium influx into the cytoplasm.

Calcium‐regulated oxidative glutamate toxicity would potentially target both cancer cells and CSCs, which results in reduced tumor relapse/recurrence rate (Chikati et al., 2018) through a novel pathway called “Oxytosis.” Oxytosis is a calcium‐mediated cell death pathway that relies on inducing oxidative glutamate toxicity (Maher et al., 2020). The critical steps that contribute to oxytosis involve glutathione (GSH) depletion, lipoxygenase (LOX) activation, and accumulation of ROS, finally leading to Ca^+2^ influx (Lewerenz et al., 2018). Store‐operated calcium entry channels (SOCE) are the calcium influx channels that regulate intracellular calcium levels (Jardin & Rosado, 2016). Interestingly, SOCE mediates calcium influx through ORAI and STIM proteins localized on the plasma membrane and endoplasmic reticulum, respectively. Under GSH depletion, LOX has activated, which in turn triggers the activation of SOCE (Roos et al., 2005). Activated SOCE triggers STIM activation, which permits the opening of the ORAI gate localized on the plasma membrane, thereby allowing calcium influx, eventually leading to oxytosis (Jardin & Rosado, 2016; Zhang et al., 2005). Figure 3 depicts the mechanism of oxytosis.

## 13 ROLE OF MITOCHONDRIAL PROTEINS IN INDUCING OXYTOSIS

Mitochondria are regarded as roots for generating ATP, is also a master regulator of cellular Ca^2+^ flux. Mitochondrial Ca^2+^ uptake is unswervingly associated with mitochondrial bioenergetics, where depletion of mitochondrial membrane potential retracts mitochondrial Ca^2+^ uptake, thereby leading to defects in the respiratory chain, which are accompanied by reduced mitochondrial ability to pump Ca^2+^. Derivatives of the TCA cycle are consecutively reduced to their respective equivalents by the electron transport chain (ETC). The proton electrochemical gradient induced by the membrane potential as a result of redox reactions, along with the pH gradient, is essential for generating ATP. Thus, the mitochondrial Ca^2+^ uptake and physiological functions must be tightly regulated by the mitochondrial membrane potential.

**Figure 4:**
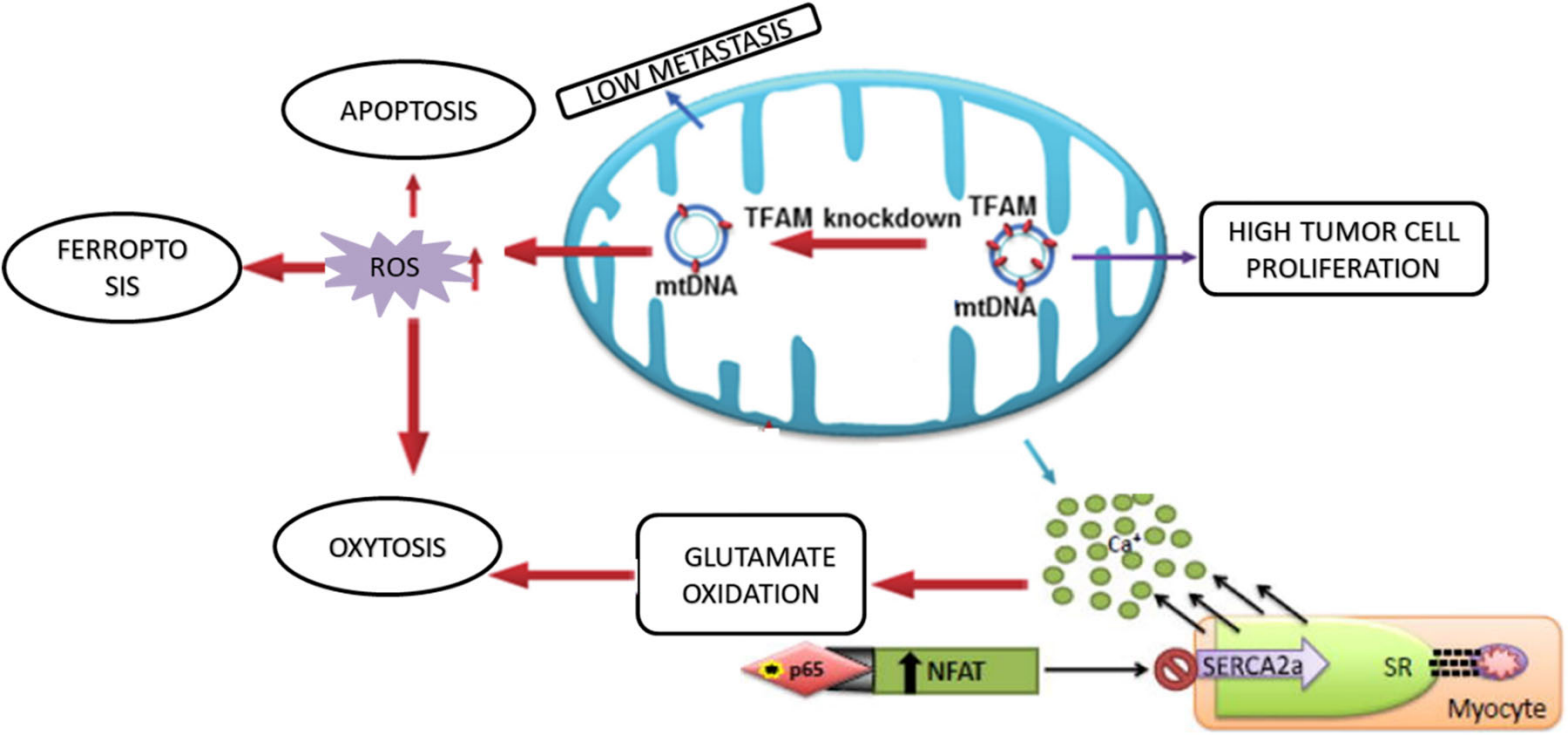
Role of Transcription factor A mitochondrial (TFAM) in inducing various cell death pathways. TFAM is one of the nuclear‐encoded mitochondrial protein that is activated by Mechanistic Target of Rapamycin. TFAM regulates tumor cell proliferation by elevating cell cycle genes. Knockout of TFAM results in induction of oxytosis by suppression of SERCA2 mediated by NFAT activation.

TFAM knockdown might result in reduced Serca2a expression localized in the nucleus, signifying that TFAM in the mitochondria monitors the nucleus to induce *Serca2a* transcription with consequent coordination of transcribed Serca2a with endoplasmic reticulum (ER) to maintain Ca^2+^ homeostasis. The ER could govern the Ca^2+^ efflux channels, thereby affecting the cytosolic Ca^2+^. Hence, low levels of ER Ca^2+^ during TFAM gene deletion in muscle might result in the decline of cytosolic Ca^2^. It has been observed that there is an increase in mitochondrial Ca^2+^ in the mice which is deficient with TFAM gene expression, suggesting that TFAM^‐/‐^ mice might induce Ca^2+^ overload in the mitochondria, thereby leading to oxidative stress (Figure 4).

TFAM might regulate Ca2+ flux by altering the mitochondrial membrane signals and their associated membrane potential to other organelles, such as the Endoplasmic reticulum and nucleus. TFAM, which is transcribed by the nucleus, regulates calcium signaling based on mitochondrial status through transmitting appropriate signals to the nucleus which is accompanied by the induction of transcription of TFAM. It has been well evident that it also prevents high‐fat diet‐induced insulin resistance.

The outcomes of TFAM inactivation play a significant role in cellular death as it is associated with loss of mtDNA, leading to mitochondrial dysfunction, which is accompanied by reduced mitochondrial membrane potential, thus arousing Ca^2+^‐dependent mitochondrial reverse signaling and thereby elevating CFAP65 (cilia and flagella associated protein 65) and PCK1 (cytoplasmic phosphoenolpyruvate carboxykinase) as observed in the study done by Woo Rin Lee et al., They observed that there is an increase in expression of CFAP65 when Ca^2+^ mediated transcription factors bind to the promoter of the *CFAP65*. Also, PCK1 expression has been stated to be governed by the CAMKII‐FoxO1 pathway mediated by Ca^2+^. Figure 2 describes the underlying mechanism of TFAM in inducing cell death.

## 14 FUTURE PROSPECTS IN CANCER TREATMENT

Conventional therapies are focused on clearing bulk tumor cells leaving behind CSCs. One of the major reasons is that since CSCs are present in G0‐phase of the cell cycle most of the chemotherapeutic drug's target only cancer cells present in the G1‐phase, S‐phase, and G2/M‐phase of the cell cycle. However, to sustain the inevitable tumor microenvironment CSC upregulates various oncogenes. For instance, ferritin levels are high in CSCs when compared with cancer cells. Similarly, expressions of mitochondrial proteins such as TFAM are highly expressed in CSCs when compared with bulk tumor cells. Hence, targeting the mTORC1 pathway via calcium homeostasis selectively kills both cancer and CSCs leading to tumor‐free environment.

Most tumors are characterized by heterogeneous phenotypes and are evolved due to multiple genetic and environmental factors, hence, inducing cell death which is different from apoptosis plays a vital role in targeting both bulk cancer cells as well as CSCs. Oxytosis is one such novel cellular death pathway that relies on the influx of mitochondrial calcium ions into the cytosol which is characterized by elevated lipid ROS, decreased Glutathione, ferritin, and elevated LOX. Therefore, focusing on the oxytosis pathway makes research one step ahead to target therapy‐resistant CSCs. Rooting evidence suggests the potential role of calcium ions in disrupting mTORC1 signaling thereby inducing oxytosis. Although numerous mTORC‐targeting drugs are currently under clinical trials, to the best of our knowledge, the potential of these drugs in the induction of oxytosis is yet to be explored. This is because oxytosis can be considered as a selective cellular death process that relies upon ferritin degradation. Hence, it will be effective on both bulk cancer cells and CSCs.

In the present review, we discussed the importance of mTOR and its role in regulating mitochondrial proteins that are essential for cancer progression, the prominent role of calcium ions in cell proliferation, and targeting calcium ion metabolism to kill both bulk cancer cells and CSCs for a tumor‐free environment. Hence, identifying the role of mTORC inhibitors in the induction of oxytosis should be focused on.

## AUTHOR CONTRIBUTIONS

**Santhi L. Pandrangi:** Wrote the manuscript; review and editing; conceptualized the study; overall supervision of the study. **Prasanthi Chittineedi:** Wrote the manuscript; figures and tables. **Ram K. Manthari:** Review and editing. **Balaji Suhruth:** Wrote the manuscript; Figures and tables.

## CONFLICT OF INTEREST STATEMENT

The authors declare no conflict of interest.
